# Is Type 2 Diabetes Mellitus a Behavioural Disorder? An Evidence Review for Type 2 Diabetes Mellitus Prevention and Remission through Lifestyle Modification

**DOI:** 10.17925/EE.2023.19.1.7

**Published:** 2023-01-11

**Authors:** Matthias Li, Mohammad Sadiq Jeeyavudeen, Ganesan Arunagirinathan, Joseph Pappachan

**Affiliations:** 1. The University of Manchester Medical School, Manchester, UK; 2. Western General Hospital, Edinburgh Centre for Endocrinology & Diabetes, Edinburgh, UK; 3. Lancashire Teaching Hospitals NHS Trust and Manchester Metropolitan University, Manchester, UK

**Keywords:** Diabetes, obesity, lifestyle modifications, low-energy diets, exercise, type 2 diabetes mellitus

## Abstract

The prevalence of type 2 diabetes mellitus (T2DM) is steadily rising worldwide due to an increasingly sedentary lifestyle combined with unhealthy food habits. Currently, the burden of diabetes on healthcare systems is unprecedented and rising daily. Several observational studies and randomized controlled trials provide clinical evidence that T2DM remission is possible by adopting dietary interventions and a strict exercise training protocol. Notably, these studies provide ample evidence for remission in patients with T2DM or for prevention in those with risk factors for the disease through various non-pharmacological behavioural interventions. In this article, we present two clinical cases of individuals who showed remission from T2DM/prediabetes via behavioural changes, especially through the adoption of a low-energy diet and exercise. We also discuss the recent advances in T2DM and obesity research, focusing on nutritional interventions and exercise and their benefits for weight loss, improved metabolic profile, enhanced glycaemic control and remission of diabetes.

Worldwide prevalence of type 2 diabetes mellitus (T2DM) is steadily increasing due to rising levels of obesity over the past 40 years caused by sedentary lifestyles and unhealthy eating habits. Diabetes now ranks ninth among the top ten causes of death worldwide, and the disability adjusted life years (DALYs) has increased more than 80% between 2010 and 2019.^1,2^ While much research has been conducted on the pharmacological management of T2DM, there is a growing body of evidence for diabetes remission without pharmacotherapy through intense behavioural interventions such as dietary modifications and structured exercise programmes. The American Diabetes Association defines diabetic remission as the maintenance of glycated haemoglobin (HbA1c) levels below 6.5% for at least three months in the absence of pharmacological intervention.^3^ Several observational studies and randomized controlled trials (RCTs) have provided sufficient clinical evidence for T2DM remission through dietary interventions.^4–7^ Multiple studies have also shown the effectiveness of nutritional and lifestyle interventions in the prevention of T2DM in individuals with prediabetes, as well as in those with normal blood glucose levels.^8–11^ All these studies provide ample evidence for remission in patients with T2DM or for the prevention of diabetes in those with risk factors for the disease largely through various non-pharmacological behavioural interventions.

The American Diabetes Association recommends the following changes in lifestyle behaviour for the prevention of T2DM: 1) intense lifestyle changes to achieve and maintain a weight loss of 7% from the initial weight and an increase in moderate-intensity physical activity to at least 150 min/week; 2) a variety of eating patterns to help patients with prediabetes; and 3) inclusion of a certified technology-assisted diabetes prevention programme as part of the management based on patient preference.^11^ These diabetes prevention programme behavioural interventions are time tested and have proven efficacy in large-scale clinical trials.^4,12^

Do these findings indicate that there is a direct link between human behaviour and the tendency to develop obesity and T2DM? Animal models have indicated that the hypothalamic neural circuitry involved in non-homeostatic feeding behaviour is activated by palatable food in the environment.^13^ Furthermore, recent data have shown that epigenetic mechanisms in response to the dietary environment promote obesity by regulating neuronal plasticity and feeding behaviour even among non-vertebrates.^14^ Human experiments also revealed hypothalamic inflammatory changes mediated through nutritional and environmental influences in individuals with obesity^15^ and showed improvement after marked weight loss through bariatric surgery.^16^ These study results again provide us with precise biomechanistic data on the role of human behavioural patterns and their influence on the development of obesity and T2DM. Therefore, with the aid of two clinical scenarios, we explore the current evidence on the human behavioural adjustments necessary to prevent T2DM or achieve remission in those with the disease.

**Figure 1:**
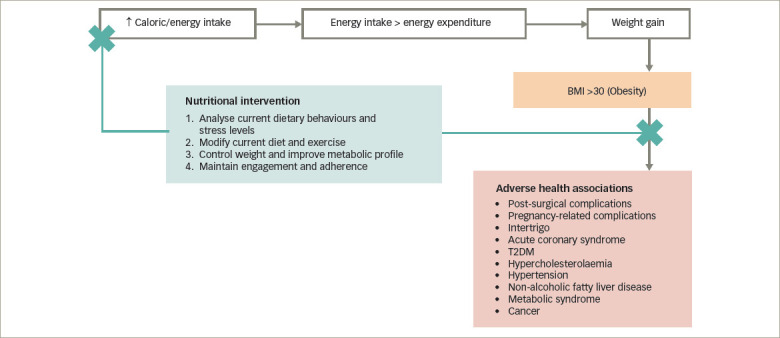
Dietary energy imbalance in the development of obesity

*BMI = body mass index; T2DM = type 2 diabetes mellitus.*

## Case 1

A 42-year-old Indian female presented to the obesity service with substantial weight gain over six years while going through serious family issues that culminated in a recent divorce. She weighed 108 kg with a body mass index (BMI) of 42 kg/m^2^. Her blood pressure was 138/92 mmHg. The biochemical investigations were: HbA1c 46 mmol/mol, total cholesterol 5.6 mmol/L, high-density lipoprotein (HDL) cholesterol 0.82 mmol/L, low-density lipoprotein (LDL) cholesterol 3.2 mmol/L and triglycerides 2.6 mmol/L, with normal thyroid and renal functions. She was offered lifestyle interventions through a dietician and exercise trainer. She adopted a 1,000 calories/day diet and started running daily to expend 300–400 calories/day. She started losing weight gradually, and after two years of regular three and six-month follow-ups, her body weight came down to 58 kg with a BMI of 24.9 kg/m^2^. Her latest biochemical profile revealed: HbA1c 35 mmol/mol, total cholesterol 4.6 mmol/L, HDL cholesterol 0.99 mmol/L, LDL cholesterol 2.9 mmol/L and triglycerides 1.7 mmol/L, with normal thyroid and renal functions.

## Case 2

An 18-year-old male presented to the diabetes clinic for management of recent-onset T2DM. His body weight was 122 kg with a BMI of 44 kg/m^2^. The biochemical profile was: HbA1c 58 mmol/mol, total cholesterol 4.5 mmol/L, HDL cholesterol 0.77 mmol/L, LDL cholesterol 3.1 mmol/L and triglycerides 3.2 mmol/L, with normal thyroid and renal functions. He was started on metformin 500 mg twice daily (increased to 1 g twice daily after a month) and entered a structured behavioural intervention programme with dietary changes and regular exercise. He kept a food diary, meticulously writing down his daily energy intake, and maintained an energy intake of around 800 calories/day. Over six months, he lost 30 kg in body weight. His investigations after six months revealed: HbA1c 42 mmol/mol, total cholesterol 4.2 mmol/L, HDL cholesterol 0.94 mmol/L, LDL cholesterol 2.6 mmol/L and triglycerides 1.9 mmol/L. Though his T2DM was in remission, he decided to continue with metformin and the active lifestyle changes he adopted as suggested by his physician.

## Dietary interventions to manage obesity and type 2 diabetes mellitus

Dietary interventions have potential benefits in improving the quality of life and the outcome of metabolic diseases, including obesity and diabetes.^17^ The fundamental principle in managing obesity and diabetes is achieving a net negative energy balance via dietary modifications. Notably, the first step in achieving negative energy balance on a sustainable and longterm basis is understanding and critically analysing the existing dietary pattern, diet preference, diet composition and the stress level of the individual.^17^ The second step is modifying the current diet, with focus on weight control and improving the metabolic profile, thus achieving the overall benefits of dietary interventions. It should be noted that the benefits of nutritional interventions emerge depending on the participants’ commitment to the interventions, the degree of adherence to the protocol and the ability to adopt the interventions on a long-term basis.^17^ Various dietary interventions are available for the management of diabetes and obesity. Of note, these interventions specifically target nutrient restriction, especially calorie restriction, without reducing the essential nutrients, avoidance of a specific food type, and modification of dietary composition or dietary patterns, including prolonged or intermittent restriction of diet.^18^ These interventions are discussed in detail below.

The key to achieving negative energy balance is modification of energy intake and expenditure through lifestyle changes.^17,19^ *Figure 1* shows a schematic representation of dietary energy imbalance in the development of obesity, with its adverse health consequences, and the nutritional adaptations required to create a negative energy balance. Specifically, a diet that provides less energy than the individuals’ actual energy expenditure creates a net negative energy balance. As dietary energy can usually be derived from several sources, including carbohydrates, fats and proteins, it is critical to consider the energy levels of the diet containing all these nutrients. On the other hand, the energy expenditure is mainly based on the resting metabolic rate, physical activity and environmental factors such as temperature, which activates the body to use energy to maintain the body temperature. As an individual’s energy expenditure is more or less the same in the resting state, most dietary interventions are targeted at reducing dietary energy intake. The energy-restricted diet could be a balanced diet with low or very-low energy (LED or VLED). There is good evidence that indicates dietary approaches, including LEDs, VLEDs and low-carbohydrate diets (LCDs), help to restore the euglycaemic status and remission of T2DM.^20^ Notably, achieving and maintaining weight loss are critical for maintaining continued remission of T2DM.^20^

### Low-energy and very-low-energy diets

Generally, low-energy diets (LEDs) aim to create an energy deficit of around 500–750 kcal/day for an individual, depending on their body weight. For example, 1,200–1,500 kcal/day is provided via diet for individuals weighing less than 113.6 kg. Similarly, 1,500–1,800 kcal/day is provided via diet for an individual weighing at least 113.6 kg. For individuals with metabolic diseases and not obesity, 800–1,800 kcal/day can be supplied via diet.^21–24^ The energy-restricted diet should be balanced with all essential nutrients, vitamins and minerals. The balanced LED may contain 45–65% carbohydrate, 20–35% fat and 10–35% proteins, vitamins, fibre and minerals.^25,26^ Similarly, very-low-energy diets (VLEDs) are developed to supply less than 800 kcal/day with all essential nutrients. Mostly, VLEDs are modified diets aimed at providing energy and 70–100 g/day of protein in the form of lean meats fortified with essential vitamins and minerals.^27^ As adopting the VLED regimen involves several risks, including cholelithiasis and dehydration, this comprehensive lifestyle modification requires medical supervision. This diet regimen is known for its rapid weight loss. Once the patient reaches the desired weight, the calorie intake is gradually increased to meet the basal metabolic requirements.

In practice, VLEDs are more effective than LEDs in reducing body weight. A meta-analysis of six RCTs indicated that LEDs resulted in a mean weight loss of 9.7% compared with VLEDs and 16.1% from initial weight over a mean of 12.7 weeks.^28^ However, the mean weight loss with LEDs and VLEDs was 5.0% and 6.3%, respectively, after one year, indicating that LEDs and VLEDs contribute lower weight loss in the longer term. In this meta-analysis, the participant attrition was almost similar, with the values ranging from 22.6–22.3% in both regimens over a mean follow-up of 29 months.^28^ Similarly, lifestyle modification and dietary interventions such as LED resulted in the loss of 6.4 kg over a period of one year. In another meta-analysis, VLEDs resulted in 10.3 kg of body weight loss over a median of 10 weeks.^29^ Along similar lines, a meta-analysis of 44 studies involving 3,817 participants who had T2DM suggested that participants who adopted a VLED of 400 kcal/day experienced a 5.4% and 17.9% weight loss at two weeks and three months, respectively.^30^ Participants on an LED of 1,200 kcal/day lost 7.3%, while those on an LED of 1,600 kcal/day lost only 2.0% after three months.^30^ Another meta-analysis of overweight or obese adults with T2DM revealed that VLEDs induced more significant weight losses at three and six months compared with other interventions, including LEDs; in patients with T2DM, VLEDs resulted in substantial weight loss.^31^

In a recent systematic review and meta-analysis assessing the efficacy and safety of LEDs and VLEDs for remission of T2DM, the authors analysed the published randomized clinical trials involving LEDs (<130 g/day from carbohydrates or <26% of a 2,000 kcal/day diet) and VLEDs (<10% calories from carbohydrates) in patients with T2DM.^32^ Interestingly, LEDs contributed to higher levels of T2DM remission (defined as HbA1c <6.5%) compared with the control diet (76/133 [57%] versus 41/131 [31%]; risk difference 0.32, 95% confidence interval [CI] 0.17, 0.47; 8 studies, n=264, I^2^=58%). Notably, patients showed significant improvements in weight loss, plasma triglyceride levels and insulin sensitivity.^32^ Moreover, in the same study, the authors observed that the VLEDs were less effective than LEDs in terms of loss of weight at six months,^32^ findings that are significantly different from an earlier meta-analysis.^28^ Analysis of the existing literature unbiasedly points towards the importance of maintaining the weight loss for continued remission, even though a euglycaemic status can be achieved with an LCD even without weight loss.^20^ Another meta-analysis examined dietary approaches to remission of T2DM and weight loss. The maximum weight loss was achieved via the energy-deficient diets containing 1.7–2.1 MJ/day (400–500 kcal) for a period of 8–12 weeks, leading to -6.6 kg (95% CI -9.5, -3.7) weight loss compared with the LEDs (4.2–6.3 MJ/day [1,000–1,500 kcal]).^33^ Overall, these studies indicate that remission of T2DM can be achieved by energy restriction and weight loss of approximately 15 kg in an individual.^34^ Energy restriction via LEDs and VLEDs can result in significant weight loss, which is essential and sufficient to reverse T2DM by restoring euglycaemia in the early years after diagnosis.^35^ Interestingly, almost 50% of people diagnosed with T2DM can stop medication for this metabolic disease and maintain euglycaemia if they follow the dietary energy restriction within the first 10 years of diagnosis.^36,37^

### Low-fat diets

Low-fat diets (LFDs) are generally adopted to reduce excess weight in patients with metabolic diseases who are obese. Typically, LFDs provide less than 30% of calories from fat and follow specifically prepared menus.^17^ Studies have indicated that LFDs are highly effective at reducing body weight and improving the standards of life in patients with obesity and metabolic diseases.^38–41^ A study involving 3,234 participants with diabetes and overweight/obesity suggested that reducing fat intake to below 25% of daily calorie requirement and restricting the food intake to 1,200–2,000 kcal/day resulted in a 7% weight loss.^38,39^ A meta-analysis indicated that LFDs significantly reduced the weight change compared with a usual diet (-5.41 kg, 95% CI -7.29, -3.54; I^2^=68%).^40^ Similar findings were also noted in another meta-analysis, where LFDs significantly lowered the initial body weight and improved the risk factors, especially the lipid profile, including total and LDL cholesterol and triglycerides.^41^

### Low-carbohydrate diets

LCDs aim to restrict the dietary carbohydrate to 60–130 g of carbohydrates per day, which is approximately ≤20–45% of daily energy intake, and to supply the remaining required energy via either fat or protein. On a very low regimen, less than 60 g of carbohydrate per day was also supplied.^17^ LCDs are preferable to LFDs as they produce significant weight loss in the short term (<6 months) and approximately the same weight loss over the long term (>12 months). Specifically, it has been shown that the LCDs induced significantly higher mean weight loss of 3.3–4.0 kg at six months compared with LFDs.^42–44^ Notably, in one meta-analysis, LCDs were shown to play a significant role in reducing body weight (weighted mean difference [WMD] -1.18; 95% CI -2.32, -0.04; p=0.04) and HbA1c (WMD -0.44; 95% CI -0.61, -0.26; p=0.00).^44^ LCDs also improved the cardiovascular risk factors and reduced plasma triglycerides (WMD -0.33; 95% CI -0.45, -0.21; p=0.00) while increasing the levels of HDL cholesterol (WMD 0.07; 95% CI 0.03, 0.11; p=0.00).^44^ Another meta-analysis on this topic also indicated the effectiveness of LCDs in losing weight and restoring the metabolic profile of participants.^45^

### Very-low-carbohydrate ketogenic diets

Very-low-carbohydrate ketogenic diets (VLCKDs) are effectively designed to induce ketosis by restricting the carbohydrate levels to less than 20 g/day during the initial period of up to 12 weeks. Subsequently, the level of carbohydrates is increased to 80–100 g/day to meet energy requirements.^46,47^ Generally, VLCKDs comprise 70%–80% fat in order to maintain ketosis and prevent gluconeogenesis from amino acids.^47^ One meta-analysis suggested that VLCKDs are very effective at inducing significant weight loss of around 10 kg in less than four weeks.^5^ Notably, the VLCKDs were highly efficient when used for more than four weeks, inducing a significant loss of 15.6 kg from the initial weight. ^5^

**Table 1:** The benefits and disadvantages of dietary interventions for weight loss and type 2 diabetes mellitus^57–66^

Dietary intervention	Benefits	Disadvantages
Magnesium and whole grains^57,58^	Reduces diabetes risk Decreases blood pressure Promotes healthy bones Reduces the frequency of migraine attacks Improves exercise performance Decreases anxiety and depression Reduces inflammation and pain Eases premenstrual syndrome	Excessive magnesium can cause magnesium toxicity
Processed red meats^59^	Great source of protein, iron, vitamin B12, zinc	Increases diabetic risk
Green leafy vegetables^60,61^	Reduces diabetes risk	No significant disadvantages reported
Walnuts^62^	Reduces diabetes risk	Can cause weight gain
Sucrose and fructose^63^	Can be substituted with other sugar up to 10% for energy consumption in patients with diabetes	Increases blood glucose
Fish^64^	Omega-3 fatty acids are healthy Improves blood pressure Reduces blood glucose levels	Increases calorie intake; Increases risk for autoimmune diseases
Fermented dairy products (e.g. yogurt)^65^	Play a role in reducing obesity, lowering body weight, reducing body fat and reducing weight gain over time	Might reduce blood pressures too much
Plant oils and tropical oils^66^	Improves glycaemic control	May raise cholesterol levels

Similarly, when VLCKDs were used for more than 12 months, they resulted in markedly higher weight loss than other dietary interventions such as energy-restricted diets.^48^ A very recent meta-analysis identified that VLCKDs effectively reduce body weight and restore euglycaemia for up to six months in patients with obesity and diabetes.^49^ Interestingly, the VLCKD improved the metabolic profile of these patients by improving triglycerides and HDL cholesterol, and reduced the requirement for antidiabetic medication up to 12 months.^49^

### High-protein diets

Generally, high-protein diets provide more than 25% of calories from protein or more than 1.6 g of protein per kg of body weight.^17^ A high-protein, low glycaemic index (HPLG) dietary intervention was shown to induce significant body weight loss (6.52 kg; 95% CI 5.50, 7.54; p<0.001) compared with controls (2.00 kg; 95% CI 0.89, 3.11; p=0.001).^50^ Similarly, the HPLG group had a significantly lower body fat percentage than the control group (p=0.002). Interestingly, the HPLG diet resulted in improvements in visceral fat, blood pressure and metabolic profile in patients with metabolic dysfunction-associated fatty liver disease.^50^ Similarly, an energy-restricted, isocaloric, high-protein, low-fat diet provided marginal benefits in terms of loss of initial body weight, fat mass and reduction in triglycerides.^51^

### Partial meal replacement and portion-controlled diets

Partial meal replacement involves one or two meal replacements fortified with all essential vitamins and minerals. Similarly, portion-controlled servings of a regular diet and meal supplements adjusted to provide the maximum permitted calories can be beneficial for controlling diabetes and obesity. Typically, a partial meal replacement plan aims to deliver a low-calorie diet containing 800–1,600 kcal/day.^52^ This is achieved by replacing regular meals with commercially available, vitamin-and mineral-enriched but energy-reduced product(s). Depending on the plan, at least one regular diet meal is provided.^52^ The meal replacements are easy to adopt, and decision-making and weightloss programmes particularly reduce overeating.^53^ Studies indicate that a portion-controlled diet that supplies 1,000–1,500 kcal/day results in significant weight losses in the range of 2.5–3.0 kg over 3–6 months, compared with a self-selected diet of conventional foods.^54^ Notably, portion-controlled servings can safely, effectively and sustainably lead to weight loss if practised over a long period.^54^ They can also improve weight-related risk factors.^52^ Data from a meta-analysis indicate that liquid meal replacements resulted in considerable reductions in body weight and improved BMI.^52^ Additionally, meal replacement helped to reduce blood pressure, body fat and waist circumference. Similarly, glucose homeostasis as estimated by HbA1c, fasting glucose and fasting insulin levels were also improved. In an RCT on portion-controlled diets, enhanced weight loss was observed during the initial period; however, this was not sustainable for a long duration, and the weight loss did not differ significantly across groups at month 6 or 12 months.^55^ Similarly, portion-controlled diets resulted in no significant weight loss in participants at six months (-18.4 lbs, 95% CI -20.5, -16.2) compared with participants on acceptance-based treatment (-15.7 lbs, 95% CI -18.0, -13.4).^56^ The different meal replacement plans seems to significantly influence weight loss, although the effects are not significantly different. *Table 1* provides a list of the different dietary interventions, as well as their benefits and disadvantages.^57–66^

### Atkins diet

The Atkins diet is a high-protein, low-carbohydrate ketogenic diet. The diet can be divided into different stages based on carbohydrate consumption. The restrictive stage of the diet limits the consumption of carbohydrates to 20 g/day; other stages limit consumption to between 25 and 90 g/day. The Atkins diet does not restrict the consumption of fat, protein or the intake of calories, and includes essential fatty acids, minerals and vitamins, and antioxidants. Robert Atkins, who developed the diet, believed that metabolic imbalances from carbohydrate consumption were the cause of obesity. The Atkins diet has gained popularity after studies proved more weight loss after the consumption of an LCD than the consumption of an LFD.

LCDs, despite being rich in fat, have been shown to reduce blood cholesterol level, including triglycerides, and increase the HDL cholesterol levels.^67^ The diet gained popularity because of its ability to treat obesity and diabetes. One study also showed that the Atkins diet increased the insulin sensitivity.^68^

### Mediterranean diet

The Mediterranean diet is based on the eating practices of Mediterranean cultures, such as in Greece and southern Italy. It consists of a high intake of minimally processed, fresh local plant foods such as fruits, vegetables, potatoes, bread, beans, nuts and seeds. It also contains a high intake of olive oil, moderate intake of dairy in the form of cheese and yoghurt, a low intake of fish, poultry and red meat, and red wine in moderation.^69^ The Mediterranean diet has been shown through systematic reviews and meta-analyses to have a significant benefit to health.^70–72^ In particular, the diet can reduce cardiovascular disease risk, obesity, cancer risk and cognitive decline, and improve glycaemic control in T2DM.^70–72^

### Intermittent fasting

Intermittent fasting is defined as the voluntary abstinence from food and drink for certain days of the week, resulting in energy deprivation.^73^ A further variation of this is time-restricted feeding, where intake is restricted to a certain number of hours in a day.^74^ Originally conducted as part of religious practices, it has now also become a popular energy-restricted diet. Intermittent fasting has been found to have significant benefits to weight loss and glycaemic control in T2DM, with the added benefit of having few restrictions around food group consumption.^75,76^ However, no significant difference in glycaemic control or weight loss was found between intermittent fasting, time-restricted feeding and other energy-restricted diets,^74–77^ suggesting that the most effective therapy would depend on patient adherence to the diet.

## Exercise interventions for the management of obesity and type 2 diabetes mellitus

**Figure 2:**
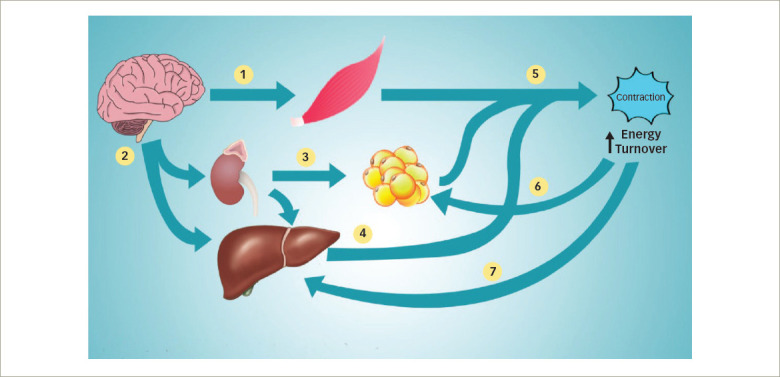
Physiological aspects of exercise metabolism and its benefit in type 2 diabetes mellitus and obesity prevention

Over the past several decades, exercise interventions have been an effective management strategy for patients with obesity and T2DM.^78,79^ Notably, the benefits of exercise are significant when combined with lifestyle, dietary and behaviour modifications.^80^ The evidence for the effectiveness of exercise is derived from multiple large RCTs. Exercise helps not only patients with obesity and diabetes but also those in the risk group for developing metabolic diseases, including diabetes and obesity.^81^ Exercise training, including aerobic, resistance and high-intensity interval training, improves the metabolic profile of patients with metabolic diseases.^82,83^ Exercise results in significant energy expenditure, which is critical for the remission of obesity-associated T2DM. Additionally, exercise training in adults with T2DM improves the overall glycaemic control, insulin action and other metabolic parameters.^82,84^ Not surprisingly, exercise training programmes also improve the functions of skeletal muscle, heart, pancreas, liver and adipose tissue.^85^ A meta-analysis provided strong evidence for the benefits of combined exercise on metabolic profile in overweight/obese patients with T2DM, including weight loss, insulin sensitivity, glycaemic control, serum lipids and blood pressure.^86^ The results were: weight loss assessed by BMI (mean difference [MD] -0.98 kg/m^2^, 95% CI -1.41, -0.56); glycaemic control assessed by HbA1c (MD -0.16%, 95% CI -0.28, -0.05); markedly reduced insulin resistance (MD -1.19, 95% CI -1.93, -0.46); reduced serum insulin levels (MD -2.18 μIU/mL, 95% CI -2.99, -1.37); reduced diastolic blood pressure (MD -3.24 mmHg, 95% CI -5.32, -1.16).^86^ Several modalities of exercises have benefits in the management of obesity and diabetes. Moderate-to-vigorous aerobic exercise training improves cardiac output while reducing the cardiovascular and overall mortality in patients with T2DM.^87^ In addition, aerobic exercise significantly induces weight loss, promotes glycaemic control, improves HbA1c levels, and improves lipid and lipoprotein metabolism.^80^ Recently, a meta-analysis indicated that structured physical exercise programmes, including aerobic training, combined training and resistance training, might improve functional capacity assessed by the 6-minute walk test (51.6 m, 95% CI 7.6%, 95.6%; I^2^ 92%), one-repetition maximum leg-press (18.0 kg, 95% CI 4.0%, 31.9%; I^2^ 0%), and maximum oxygen consumption (2.41 mL/kg min, 95% CI 1.89%, 2.92%; I^2^ 100%) in patients with T2DM.^88^ Interestingly, a recent study also indicated that a virtual multidisciplinary intensive lifestyle intervention was as effective as in-person intervention programmes aimed at increasing body weight loss, improving metabolic profile and reducing medication in patients with T2DM and obesity.^89^ *Table 2*^90–93^ summarizes the health benefits and safety issues associated with various exercise programmes, and *Figure 2* summarizes the physiological aspects of exercise metabolism and how they benefit T2DM and obesity prevention.

### Tai chi

Tai chi is an ancient tradition from China that is practised as a form of exercise. It involves a series of focused slow movements along with deep breathing and stretching. It involves constant body movements, with no pauses as the body changes from one posture to the other.^94^ Tai chi is known as an effective way of managing blood glucose levels and HbA1c in patients with T2DM.^95^ A recent meta-analysis showed that long-term practice of tai chi significantly lowers blood glucose levels and HbA1c in patients with T2DM.^96^ Another meta-analysis including 798 individuals showed that tai chi is more efficient at lowering blood glucose levels (MD -1.39, 95% CI -1.95, -0.84; p<0.0001) and HbA1c (MD -0.21, 95% CI, -0.61, 0.19; p=0.31) compared with no exercise.^97^ No significant difference was observed between aerobic exercises and tai chi in lowering blood glucose levels (MD -0.50, 95% CI -1.02, 0.02; p=0.06), and although tai chi showed a greater reduction in HbA1c than aerobic exercise, the statistical significance was only borderline (MD -0.19, 95% CI -0.37, 0.00; p=0.05).^97^ These data indicate the potential benefits of tai chi on the management of metabolic diseases.

### Yoga

Yoga originated in India. It is considered to bring balance and harmony between the mind, body and emotions.^98^ Yoga is practised worldwide for the management and control of lifestyle diseases.^99,100^ T2DM is one of the lifestyle diseases that can be controlled using yoga. Yoga draws on immune mechanisms along with psycho-neuro-endocrine mechanisms to produce a beneficial effect in the treatment of T2DM.^101,102^ The incorporation of yoga into the daily routine is also known to reduce the probability of people without diabetes from becoming diabetic and to reduce the risk and complications associated with T2DM.^101,102^ Clinical studies have demonstrated the use of yoga in lowering blood glucose levels and reducing the incidence of T2DM.^103,104^ Yoga helps in the activation of anti-stress mechanisms along with parasympathetic activation, aiding lipid metabolism, improving glucose tolerance and increasing the sensitivity to insulin, and hence, the overall psychological and metabolic profiles of patients.^105,106^ The practice of yoga includes various asanas, mudras, meditation, pranayamas and bandhas that can lower blood glucose levels, as well as control and manage various comorbidities associated with T2DM; thus, yoga is a useful method of T2DM management.^107^

### Swimming

Many studies have proved that exercise more than three times a week at 55–90% of maximum heart rate is necessary to enhance the physical strength of individuals with obesity and overweight.^108,109^ Aquatic exercises such as swimming can reduce body fat with enhanced stability compared with other ground exercises. A study conducted by Lee et al. in 2014 showed that regular swimming significantly reduced fat percentage and fat-free mass, and significantly enhanced physical fitness, muscular endurance, flexibility and right leg vascular compliance.^110^ Although aquatic exercises improve blood glucose level, physical fitness and overall cardiovascular health, it is recommended to not overdo the exercise or practice when feeling nauseous, fatigued, dizzy, weak, etc.^110,111^ Interestingly, swimming could be a potent ameliorator for insulin resistance in T2DM, via the Wnt3a/β-catenin pathway.^112^

**Table 2:** The health benefits and safety issues of exercise programmes for weight loss and type 2 diabetes mellitus

Exercise programme	Health benefits	Associated safety issues
Aerobic exercise^90,91^	Improves insulin sensitivity Improves blood glucose control Improves fat oxidation and storage in muscle Reduces blood pressure	Without the use of exogenous insulin or insulin secretagogues, the risk of exercise-induced hypoglycaemia is negligible
Resistance exercise^92^	Enhances skeletal muscle mass Reduces low-density lipoprotein cholesterol	Effects not reported significantly so far
High-intensity interval training^93^	Reduces blood glucose Increases insulin secretion	May cause substantial depletion of muscle glycogen, thereby increasing risk for postexercise hypoglycaemia in users of insulin or insulin secretagogues

*1. Motor cortex signals muscle contraction; 2. Sympathoadrenal system activates; 3. Non-esterified fatty acids (NEFAs) mobilize from adipose tissue; 4. Liver converts glycogen store to glucose; 5. Energy supplied for muscle contraction; 6. NEFA uptake increases and myokines activate to brown adipose tissue; 7. Hepatic glucose production enhances for activity.*

## Weight-loss programmes

The largest commercial weight-loss programme in the USA, WW International (formerly Weight Watchers International), provides weight loss services and guidelines to worldwide users. Behaviourally oriented WW programmes mainly concentrate on diet modification, physical exercise, behaviour modification and group support to achieve weight loss. WW supports a nutritionally balanced diet with a moderate energy deficit and promotes regular exercises for a 1–2 lb weight loss per week. WW also encourages the development of behavioural skills such as selfmonitoring and goal setting, and conducts open-group meeting sessions of 30–45 minutes.^113^ A study conducted in the UK and funded by WW included 29,326 National Health Service referrals to the WW programme for weight management.^114^ Participants achieved a median weight loss of 2.8 kg, and a third of participants achieved a 5% weight loss.^114^ Another study, conducted by Holland-Carter et al. in 563 individuals, showed that WW participation improved psychosocial outcomes specific to T2DM and obesity.^115^ In 2017, WW participation reduced the risk of T2DM in half of the participants, and blood glucose levels returned to the normal range.^116^

## Areas of uncertainty and emerging research questions

Dietary interventions are highly beneficial in the remission of diabetes and improved obesity during the period of intervention.^17^ However, there is a risk of recurrence if the intervention is discontinued. Studies are required to understand the phenomenon and devise novel strategies to maintain the glycaemic status and body weight even during non-adherence to the interventions. Different dietary interventions and exercise programmes share potential benefits in improving the quality of life in patients with diabetes and obesity. It is unclear how much dietary intervention and training are essential to keeping individuals healthy with improved weight loss and metabolic profile. It is crucial to optimize the nutritional interventions and exercise regimen to avoid tissue injury, especially muscular and bone injury in diabetes and obesity, and to reduce the risk of cardiovascular complications in asymptomatic patients with preexisting cardiac diseases.

Additionally, the effect of exercise training programmes and their link with dietary interventions needs to be studied in patients with obesity and diabetes. Investigation into whether dietary interventions have potential additive effects with exercise regimens will inform further development in management strategies. The current findings indicate the benefits in maintaining glycaemic control for patients with diabetes and in reducing risk factors for cardiovascular diseases.^20,80^ However, insufficient evidence is available regarding remission of severe complications of diabetes such as retinopathy, nephropathy and neuropathy. Future research is required to understand the impact of exercise and dietary interventions in reducing the pathogenesis of these complications in patients with metabolic diseases. The positive and negative interactions between exercise, lifestyle modifications and medications are not clear at this stage. Future works are required to elucidate the associations and complications between these factors in patients with obesity and diabetes.

The biggest problem with behavioural interventions is the difficulty of maintaining adherence to achieve long-lasting and sustainable benefits. Moreover, there are no long-term data on such interventions because of the difficulty in performing such studies due to high attrition rates.^117,118^ The inherent inability of human beings to maintain such behavioural adaptations, possibly augmented by various daily life stressors, results in relapse of T2DM from weight regain. Such non-responders become discouraged and ‘shut down’ due to weight regain, even after an initial response.^119^ Therefore, although we should attempt behavioural interventions for remission of the disease in all patients with new-onset T2DM and prediabetes, we should also be realistic about the high risk of failure or relapse in these individuals.

## Conclusions

In this review, we presented two case scenarios showing remission from/ prevention of T2DM and improved quality of life after adopting dietary interventions, especially the VLCD, and exercise regimens. Notably, both the patients attained their diabetes/prediabetes remission with marked improvements in their metabolic and lipid profiles. We have also reviewed the recent literature on the benefits of various dietary interventions and exercise in improving the quality of life in patients with diabetes and obesity. Overall, the data from our cases and others have undoubtedly pointed to the possibility of using these interventions for the remission of diabetes and maintenance of glycaemic control. Therefore, although we cannot label T2DM as a behavioural disorder, we can clearly state that the disease is largely preventable by intense behavioural interventions, and possibly that a significant proportion of patients with T2DM can attain diabetes remission by massive changes in their lifestyles through behavioural modifications. However, we should also be mindful of the high risk of failure of these interventions and the chance of relapse after initial success.

**Figure 3:**
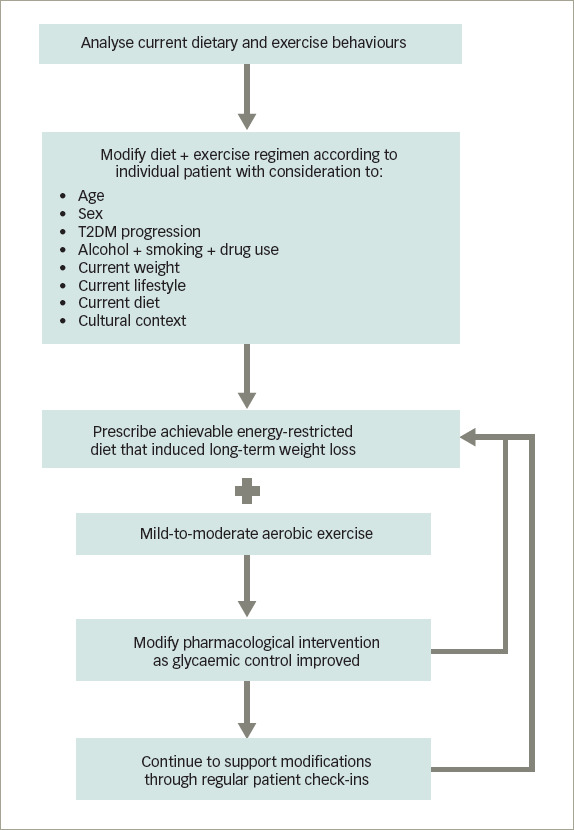
Algorithm to modify the risk of type 2 diabetes mellitus and potentially reverse the disease at its early stage

*T2DM = type 2 diabetes mellitus.*

*Figure 3* shows an algorithm designed to modify the risk of T2DM and potentially reverse the disease at its early stage.
